# Autosomal dominant hereditary ataxia in Sri Lanka

**DOI:** 10.1186/1471-2377-13-39

**Published:** 2013-05-01

**Authors:** Dulika S Sumathipala, Gayan S Abeysekera, Rohan W Jayasekara, Chantal ME Tallaksen, Vajira HW Dissanayake

**Affiliations:** 1 Human Genetics Unit, Faculty of Medicine, University of Colombo, Colombo, Sri Lanka; 2 Department of Neurology, Oslo University Hospital, Oslo, Norway; 3 Faculty of Medicine, Oslo University, Oslo, Norway

**Keywords:** Ataxia, CAG repeats, Depression, Dominant, Genetics, Hereditary, INAS, SARA, Spinocerebellar

## Abstract

**Background:**

Spinocerebellar ataxias (SCA) are a group of hereditary neurodegenerative disorders. Prevalence of SCA subtypes differ worldwide. Autosomal dominant ataxias are the commonest types of inherited ataxias seen in Sri Lanka. The aim of the study is to determine the genetic etiology of patients with autosomal dominant ataxia in Sri Lanka and to describe the clinical features of each genetic subtype.

**Methods:**

Thirty four patients with autosomal dominant ataxia were recruited. For every patient the following was done: recording of clinical details and genotyping for SCA 1, 2, 3, 6, 7, 8, 12, and 17.

**Results:**

Sixty one per cent of the subjects were identified as SCA1. One subject had SCA2, 12 remain unidentified. Mean age at onset was 34.8 ± 10years for SCA1 and 32.7 ± 9.8 for non SCA1. 76% of SCA1 patients and 50% of non SCA1 were using walking aids. Quantification of symptoms and signs were similar in the SCA1 and non SCA1 groups. Clinical depression was evidenced in 68.4% of SCA1 and 75% non SCA-1 patients. Mean CAG repeat length in SCA1 patients was 52.0 ± 3.8, with greater anticipation seen with paternal inheritance.

**Conclusion:**

SCA1 was the predominant subtype and showed similar phenotype to previous reports. However, disease severity was higher and depression more prevalent in this population than previously described.

## Background

Spinocerebellar ataxias (SCA) are a clinically and genetically heterogeneous group of neurodegenerative disorders [1]. Clinical features include progressive limb and gait ataxia, loss of coordination, disturbance of speech and occulomotor control. Patients may also present with additional non ataxia symptoms such as pyramidal (hyperreflexia, extensor plantar response, spasticity), extrapyramidal (myoclonus, rigidity, chorea, dystonia,) and autonomic dysfunction. With clinical phenotypes variable and often overlapping, genotyping has emerged as a more accurate classificatory tool. At present, more than 30 different SCA subtypes have been genetically defined with 22 subtypes having genetic mutation isolated, with 12 subtypes being caused by repeat expansion [2].

Wide geographical variation in prevalence of autosomal dominant SCA is seen partly due to founder mutations [3]. SCA 1, 2, 3, 6, and 7 caused by trinucleotide repeats are the most prevalent SCA subtypes worldwide. SCA1 and 2 are common in Italy, South Africa and India. SCA 3 is the most prevalent subtype in Portugal, Germany, Brazil, and China. High frequency of SCA 6 is reported from Japan, Germany and North American countries. SCA7 is reported as a common SCA type of South African families of Black ethnic origin [4]. The majority of epidemiological studies conducted on the prevalence of SCA are from Europe. Only a few have focused on Africa and Asia. In the South Asian region, most studies originate from India, and show a variation in SCA prevalence with increased SCA 1 in south India and SCA 2 in east India [5-9] (Table 1).

**Table 1:** Reports of SCA subtypes in the Indian Subcontinent up to 2012

**Region**	**Year**	**N**	**SCA subtype**				
			**SCA 1**	**SCA 2**	**SCA 3**	**SCA 6**	**SCA 7**
India South [5]	2003 -2006	284	34 (31.8%)	24 (22.4%)	15 (14%)	N/A	N/A
India – South [6]	2005	236	7.2%	N/A	N/A	N/A	N/A
India East ( West Bengal ) [7]	2004	28	2 (14.3%)	4 (28.6%)	5 (35.7%)	0	0
India Mostly North [8]	2000	42	3 (7.1%)	10 (23.8%)	2 (4.8%)	0	0
India East ( West Bengal ) [9]	1997 – 1999	57	6 (10.5%)	10 (17.5%)	4 (7.0%)	1 (1.8%)	0

N = patient number included in the study.

N/A = these subtypes were not investigated in the studies.

Genetic diagnosis of familial disease remains a relatively new field in Sri Lanka. Rare diseases like hereditary ataxia may remain undiagnosed due to acceptance of the disease as a familial idiosyncrasy. This is particularity true if it is characterized by late onset and slow progression. Knowledge about the frequency, occurrence and clinical type of disease is an important public health care issue, necessary for identification, follow-up and genetic counseling of patients.

To the best of our knowledge there are no data regarding SCA in Sri Lanka, where a population of approximately 20 million reside. We report the clinical and genetic results in 34 patients identified with autosomal dominant ataxia in Sri Lanka. Though not representative for the whole of Sri Lanka, this study was conducted as an initial assessment of a cohort of patients for the presence of autosomal dominant ataxia in Sri Lanka, and to further characterize the phenotypes and genotypes of the identified affected subjects.

## Methods

This study was conducted at the Human Genetics Unit (HGU), Faculty of Medicine, University of Colombo, which is the only center in Sri Lanka providing genetic diagnostic and counseling services in the government sector. It receives referrals from all the tertiary care hospitals and other healthcare institutions across Sri Lanka.

The archives of the Human Genetics Unit were accessed for referrals of dominant ataxia between January 2005 and December 2011. Of the total 40 patients in the referred database 22 were contactable and agreed to participate in the study. A further patient population was identified from an isolated geographical region following information from the already included patients. Twelve patients were recruited from this region.

Inclusion criteria were: Adults aged 18 years and more with progressive cerebellar ataxia with autosomal dominant inheritance pattern of affected family members and/or verified molecular diagnosis. Exclusion criteria were: ataxia with secondary cause or sporadic ataxia. Thirty-four patients were thus included in the final evaluation.

### Clinical examination

The patients were examined and evaluated by 3 investigators of the project. They were evaluated according to the standard clinical protocol for ataxia patients (SPATAX evaluation form )[10], which carries a score of 0 to 7, with 0 = no functional handicap and 7 = confined to bed. Scale for Assessment and Rating of Ataxia (SARA), which quantifies ataxia from 0 to 40 and Inventory of Non Ataxia Symptoms (INAS), which is a semi-quantitative variable of extracerebellar involvement summed into 16 binary variables [11,12]. Coexistent depression was assessed using the Patient Health Questionnaire (PHQ) [13]. Correlations between disease phenotype and genotype and depression levels were assessed.

The study protocol was approved by the institutional ethics committee of the Faculty of Medicine, University of Colombo, Sri Lanka. All patients gave written informed consent prior to participating in the study.

### Molecular analyses

DNA was extracted from peripheral blood leucocytes. Genetic testing for SCA 1, 2, 3, 6 7, and 8 repeats was done by multiplex amplification and capillary electrophoresis [14,15] These analyses were performed at the Human Genetics Unit, Faculty of Medicine, University of Colombo, Sri Lanka. In addition SCA 12 and SCA 17 targeted mutation analyses of trinucleotide repeat regions were performed according to standard earlier published protocols at the research diagnostic section of the Department of Medical Genetics, Oslo University Hospital [16,17].

### Statistics

Descriptive statistics were performed using percentages, means and standard deviations. Comparison of non parametric data between groups was done using Chi square test. Comparison of means was done using the Students’ t test with significance observed using ANOVA. Correlations were obtained using the Pearson correlation. Binary and continuous variables were analyzed using logistic regression and multiple regression analysis respectively. Statistical significance was set at p < 0.05.

## Results

A total of 34 patients were recruited from 27 families with autosomal dominant hereditary ataxia. Spinocerebellar ataxia type 1 (SCA 1) was found in 14 families. SCA 2 was found in 1 while no genetic diagnosis could be established in the remaining 12. SCA type 3, 6, 7, 8 12 or 17 mutations were excluded. A SCA1 genotype frequency of 51.8% (14/27) was thus established in the cohort making it the predominantly observed SCA subtype.

Mean age of onset of disease was similar in SCA1 and non SCA1 patients; duration of disease was longer in the non SCA1 patients. Patient disability and SARA scores were higher in the SCA1 group (Table 2). The mean SARA score of SCA1 patients had positive correlation with disease duration (R^2^ = 0.2, p = 0.2) and negative correlation with the age at onset (R^2^ = 0.6, p < 0.05). The most prevalent non-ataxia signs at examination seen in SCA1 and non SCA1 patients were hyperreflexia (76.2% and 67%) and spasticity (81% and 50%). However among the non- ataxia symptoms non SCA1 patients had more sensory symptoms such as reduced vibration sense (76.2% vs 50% and 28.6% vs 41.7%). Hyperreflexia, spasticity, urinary dysfunction and cognitive decline increased significantly with age in the SCA1 patient group alone.

**Table 2:** Clinical characteristics of patients with SCA1, SCA2 and unknown genetic etiology

**Genetic Diagnosis**	**N**	**Mean age symptom onset (years)**	Disability level*	SARA**	INAS***	Clinically relevant depression *****
				**(0–40)**	**(0–16)**	
SCA1	21	34.8 ± 10	4 ± 2	18.8 ± 9.7	3.6 ± 2.4	68.4%
SCA2	1	34.0	6	26	6	100%
Unknown genetic etiology	12	32.7 ± 9.8	3 ± 2.7	13.7 ± 7.1	3.8 ± 2.1	75%

N = patient number included in the study.

*Disability level was quantified using the SPATAX score. (0 = No functional handicap, 1 = No functional handicap but signs at examination, 2 = mild unable to run, walking unlimited, 3 = moderate unable to run, limited walking without aid, 4 = severe walking with one stick, 5 = walking with two sticks, 6 = unable to walk requiring wheelchair, 7 = confined to bed).

**SARA = Scale of Assessment and Rating of Ataxia.

***INAS = Inventory of Non ataxia Symptoms.

****Clinically relevant depression as percentage of total subtype patient population - assessed using the Patient Health Questionnaire 9 (PHQ9).

The PHQ was answered by 19 (90.5%) patients with SCA1 and 100% non- SCA1 patients. There was a high, clinically relevant level of depression in both groups (68.4% and 75%). Sleep disturbance was cited as the most significant symptom in both patient groups.

The mean CAG repeat length in SCA 1 patients was 52.0 ± 3.8 (47–59). Anticipation was present in the patient group with a significant reduction in the age of onset of patients compared to their parents (p < 0.005). There was also an inverse correlation between the age of onset and CAG repeat length in the expanded allele. Most subjects had inherited their mutation paternally (71.4%) with a significant difference in the degree of anticipation seen according to the transmitting parent (Table 3).

**Table 3:** Familial inheritance pattern and genetic characteristics of SCA1 patients

**N**	**SCA1 = 21**	
	**Father**	**Mother**
Parental transmission – Father / Mother	15 (71.4%)	6 (28.6%)
Age difference between transmitting parent and child – (Mean ± SD)	12.6 ± 5.4	6.8 ± 2.4
CAG repeat number - Mean ± SD (range)	52.0 ± 3.8 (47–59)	

N = Number of patients.

**Figure 1:**
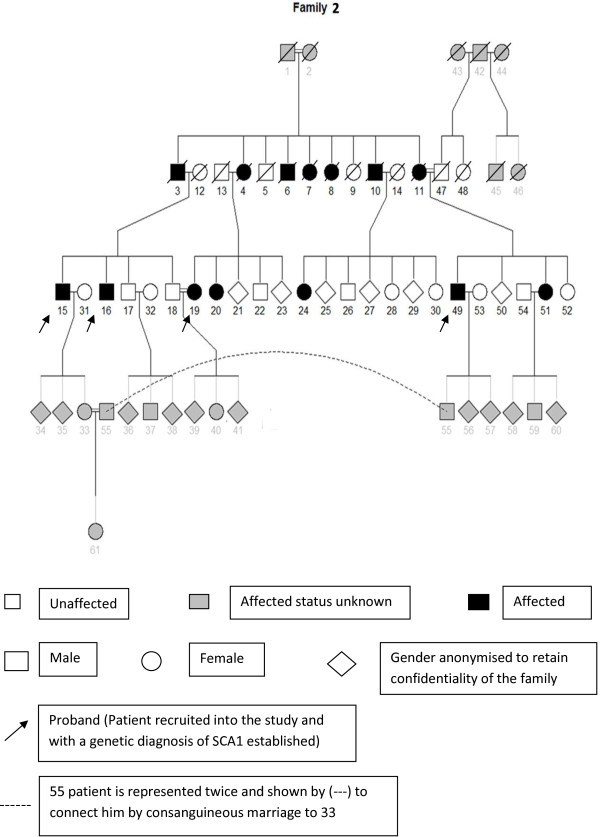
Pedigree of a family with SCA 1 from the Southern province of Sri Lanka.

Of the total 21 SCA1 patients, 15 were from a single geographical location in the southern province. A large pedigree in the study was from this region (Figure 1). It accounts for 4 of the 15 patients recruited into the study. (Patient number 15, 16, 19 and 49).

## Discussion

This is the first study in clinical genetics of dominant ataxia conducted in Sri Lanka. Interestingly, SCA 1 was the most frequent occurring type of SCA identified in the study, accounting for 61.7% of all identified SCA. Only one SCA 2 family was found, but no SCA3, 6, 7, 8 12 or 17. Moreover, the clinical findings suggest a more severe disease course in the SCA 1 patients compared with previous reports. Last but not least this study revealed the presence of isolated non diagnosed patients’ groups in the country.

### Study population

The patients recruited were predominantly from the south and west of the country which accounts for approximately 43% of the total population [18]. This recruitment bias makes it impossible to give a reliable epidemiological data for the whole country. However when comparing the SCA subtype found in Sri Lanka with Indian populations (Table 1), the similarities are more with South Indian data. The geographical and genetic proximity with South India with migratory phenomena may have influenced the epidemiology of SCA in Sri Lanka [5,19]. Further studies are needed to confirm the true epidemiological spread of SCA subtypes and haplotype analysis to investigate the presence of founder mutations.

Apart from the 12 patients recruited from the South all other patients were recruited based on recall of archives. This may also have caused a recruitment bias by excluding patients from remote regions and patients with SCA phenotypes that include non-ataxia symptoms such as cognitive impairment, emphasizing the need a further epidemiological study.

### Clinical phenotype

Clinical findings in the SCA1 patients are in accordance with most published series. Anticipation is present with a significantly higher contribution of mutant alleles paternally transmitted in the study population. This is in accordance with the previous findings that in SCA 1, CAG repeats are more unstable and larger in size when transmitted paternally [20].

An increase in disability level with duration of disease was present as expected; however the level of disability was greater than compared to previous reports. Although the disease duration was shorter in our study than in EUROSCA (7.4 ± 3.1vs 9.5 ± 5.5) the mean SARA score of 18.8 ± 9.7 was higher (15.6 ± 9.1) [21]. This may be related to the relatively higher CAG repeat length in the study population (Tables 2 and 3), resulting in a rapidly progressive severe phenotype. In addition a poor level of para-clinical supportive services such as physiotherapy for the neurodegenerative disease might also be contributing to disability.

INAS count showed increased pyramidal signs which were similar to previous reports on SCA1. However the mean INAS score was lower than previously reported (3.6 ± 2.4 vs 5.0 ± 2.3) indicating a phenotype with relatively less extracerebellar signs [22]. This may of course be a bias due to the sample’s small size.

There were a surprisingly higher number of patients with depression compared to previous reports that used the same scoring system in SCA patients (68.4% vs 24.5%) [13]. Between the two studies the proportion of patients with no depression, mild, moderate and severe depression was inversely related, with high patient numbers with severe depression in this study population. There was significantly less social support, financial security and increased disability due to lack of physiotherapy in our patients’ cohort. Identification and treatment of depression was also nonexistent. In chronic neurodegenerative disease physiotherapy and occupational therapy have been shown not only to slow the progression of neurological deterioration but also to have positive effects on the mental health status of patients as does medical interventions for depression [23,24]. This highlights the need for supportive care in the hereditary ataxias.

### Familial aggregation of mutation

A familial aggregation of SCA1 was found in the Southern region of Sri Lanka. The presence of a founder mutation could account for this, and further studies are ongoing to verify this hypothesis. Surprisingly consanguinity was found in SCA 1 families. It appears to be induced by the social conventions [25]. It is part influenced by the inter marriage of individuals within the same caste and by the need to limit the burden of social stigma attached to the disease on the individual by marriage into a similarly affected family. The need of genetic services and health information and education to these regions is clearly emphasized by these findings. The researchers travelled to the homes of the patients in this village for examination and investigations. Until recruitment to the study they had not been seen by a medical professional. This may indicate that further pockets of isolated patients’ populations are present within the island. The single SCA2 patient identified was the only patient from the central region. The learning’s of this study are that other patients with SCA2 may be present in this region and should be specifically looked for by health care workers in order to provide a genetic diagnosis.

## Conclusion

This is the first report on autosomal dominant in Sri Lanka, with mostly SCA1 patients in the cohort of patients studied. There are, however, indications that more affected subjects are present in hidden pockets, isolated and undetected. Further studies are needed to identify the epidemiological spread of patients and confirm founder mutations. High levels of clinically significant depression indicate that health care services need to focus in this area to increase the quality of life in affected subjects.

## Competing interest

The authors declare that they have no competing interests.

## Authors’ contributions

DS contributed to the clinical and molecular genetic studies, preformed the statistical analysis and drafted the manuscript. GA carried out the molecular genetic studies. RW helped draft the manuscript. CT participated in the design and coordination of the study and helped draft the manuscript. VW conceived the study, participated in the study coordination and helped draft the manuscript. All authors read and approved the final manuscript.

## Pre-publication history

The pre-publication history for this paper can be accessed here:

http://www.biomedcentral.com/1471-2377/13/39/prepub
